# Efficient Synthesis and Reaction Kinetics of Readily Water Soluble Esters Containing Sulfonic Groups

**DOI:** 10.3390/molecules191221022

**Published:** 2014-12-15

**Authors:** Krzysztof R. Idzik, Karsten Nödler, Friedrich Maier, Tobias Licha

**Affiliations:** Department Applied Geology, Geoscience Centre of the University of Göttingen, Goldschmidtstrasse 3, Göttingen 37077, Germany

**Keywords:** sulfonic acid esters, primary alcohols, phenols, acylation, reaction kinetics

## Abstract

A series of various readily water soluble esters were synthesized by a very efficient procedure. These compounds can be useful as thermosensitive tracers for studying the cooling progress in a low enthalpy georeservoir exploitable by double flash geothermal power plant systems. The kinetics of their hydrolysis was investigated. Acylation of primary alcohols or phenols was carried out by a method based on a single-phase solvent system consisting of ethyl acetate acting as an organic solvent and triethylamine acting as a catalyst. Products were characterized by ^1^H-NMR, and ^13^C-NMR.

## 1. Introduction

Compounds susceptible to undergoing hydrolysis have raised interest as thermosensitive tracers in geothermal applications [1,2]. By knowing their kinetic hydrolysis parameters it becomes possible to track thermal fronts in geothermal reservoirs and thus, predict the thermal drawdown of the georeservoir over time. Recently, the underlying theory of their applicability in georeservoirs has been verified [3]. The biggest advantages of these new tracers are their high water solubility due to free sulfonic groups, an absence of geogenic background concentrations, and no fluorescence emission from esters, while at least one of the hydrolysis reaction products shows fluorescence. The latter allows performing online measurements under *in situ* conditions further reducing operational costs. While amides [4] allow the study of high enthalpy systems and have long residence times, esters [5] are especially useful to study low enthalpy systems or to conduct short term experiments such as push-pull experiments. 

Compounds with sulfonic groups also play an important role in electrochemistry. Among others, Zengbin [6] showed that sulfonated aromatic molecules exhibited strong adhesion and chemical stability, and lithiated fluorinated sulfonic side chains help to enhance the ionic conductivity and Li+ ion diffusion due to the charge delocalization over the sulfonic chain. Aliphatic esters containing sulfonic groups are further commonly used as plasticizers for PVC plastisols [7].

Eba *et al.* developed a method for the solid-phase detection of phospholipase A2 (PLA2) based on 1-octanoyloxynaphthalene-3-sulfonic acid, which was found to be a good substrate of PLA2. The substrate is hydrolyzed by PLA2 into 1-naphthol-3-sulfonic acid, which is spontaneously coupled with a coexisting diazonium salt and forms a red-purple azo dye [8].

Acylation provides an inexpensive and efficient way for obtaining esters in a synthetic process [9]. A number of reagents can be used for carrying out this reaction, such as benzoyl chloride, primary alcohols, phenols, and carboxylic acids.

There are plenty of methods for obtaining esters, for example a Fischer esterification, which involves treating a carboxylic acid with an alcohol in the presence of polymeric sulfonic acids as a catalyst and a dehydrating agent sequestering water. Water can also be removed by distillation as a low-boiling azeotrope by means of toluene in conjunction with a Dean-Stark apparatus. The Steglich esterification is a method of forming esters under mild conditions. This method is popular in peptide synthesis, where substrates are sensitive to harsh conditions such as high temperatures. Dicyclohexylcarbodiimide is used to activate the carboxylic acid for further reaction and 4-dimethylaminopyridine is used as an acyl-transfer catalyst [10].

A further method to obtain esters is the reaction of alcohols or phenols with acyl chlorides. The analogous acylations of amines result in the formation of amides. Esters can also be obtained by transesterification, carbonylation, and alkylation of carboxylate salts [11]. Very useful methods to obtain esters are the Favorskii rearrangement of α-haloketones in the presence of a base [12], the Baeyer-Villiger oxidation of ketones with peroxides [13,14], and the Pinner reaction of nitriles with an alcohol [15].

## 2. Results and Discussion

**Scheme 1:**
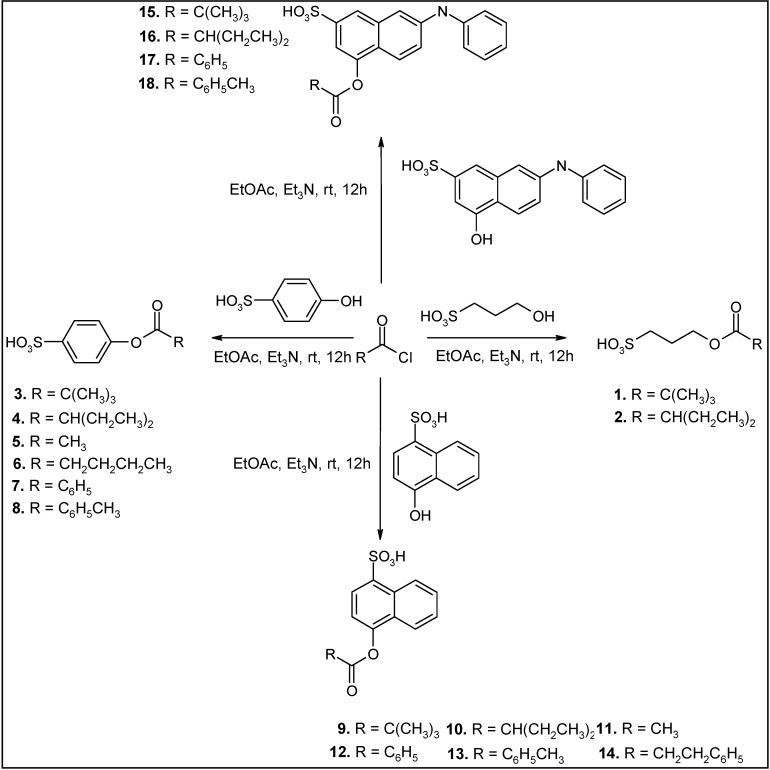
Synthesis of esters containing sulfonic acid groups.

The main problem we encountered in our study was a poor to very poor solubility of the starting materials in typical organic solvents, especially those with sulfonic groups. For example, during the Fischer esterification conducted with toluene in conjunction with a Dean-Stark apparatus a suspension was formed, which prevented a good water separation. This well-known method described above has proved to be useless for the preparation of esters having a sulfonic group. Consequently, we had to find an alternative method for obtaining water soluble esters containing sulfonic groups. In acetonitrile all reagents were completely soluble and it was used in the reaction as a substitute for toluene. Triethylamine was used as a base. Unfortunately, we did not observe any product formation. Acetonitrile was replaced by ethyl acetate with good results. We noticed that long reaction times and an insufficient amount of triethylamine could lead to product hydrolysis, in particular in the acidic environment as a result of an acid chloride hydrolysis. The highest yields were obtained after reaction times of 12 h with excess of triethylamine. By applying this method it is possible to obtain products in a fast way and in very good yield. The products are crystallized from methanol–water solution or purified by column chromatography (Scheme 1). The results are summarized in Table 1.

**Table 1:** Acylation of primary alcohols and phenols.

Entry	Reagent 1 (R_1_-COCl)	Reagent 2 (R_2_-OH)	Product (R_1_-COO-R_2_)	Yield (%)
1	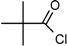	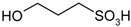	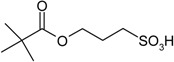	85
2	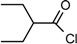	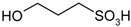	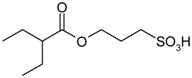	80
3	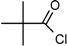	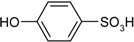	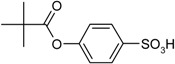	99
4	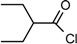	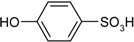	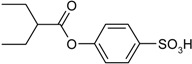	96
5	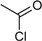	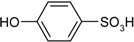	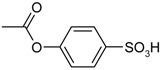	92
6	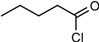	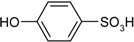	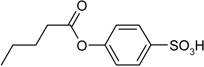	86
7	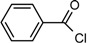	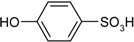	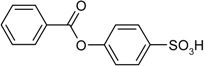	98
8	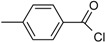	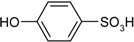	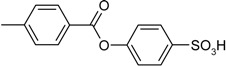	96
9	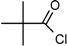	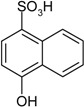	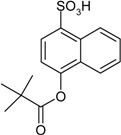	99
10	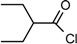	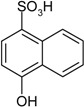	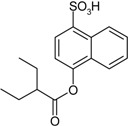	95
11	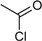	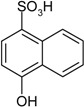	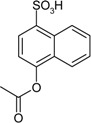	99
12	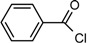	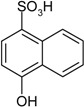	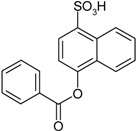	98
13	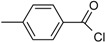	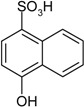	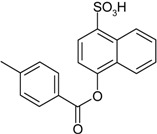	96
14	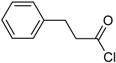	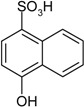	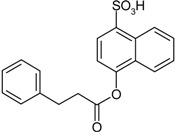	40
15	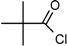	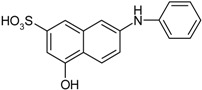	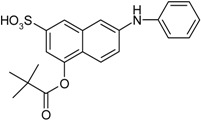	80
16	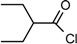	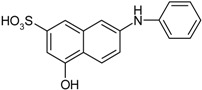	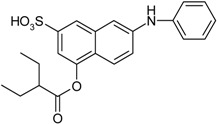	78
17	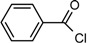	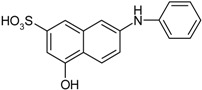	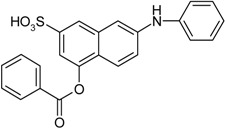	85
18	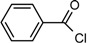	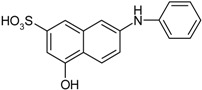	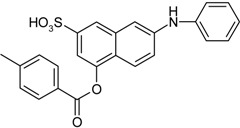	82

**Figure 1:**
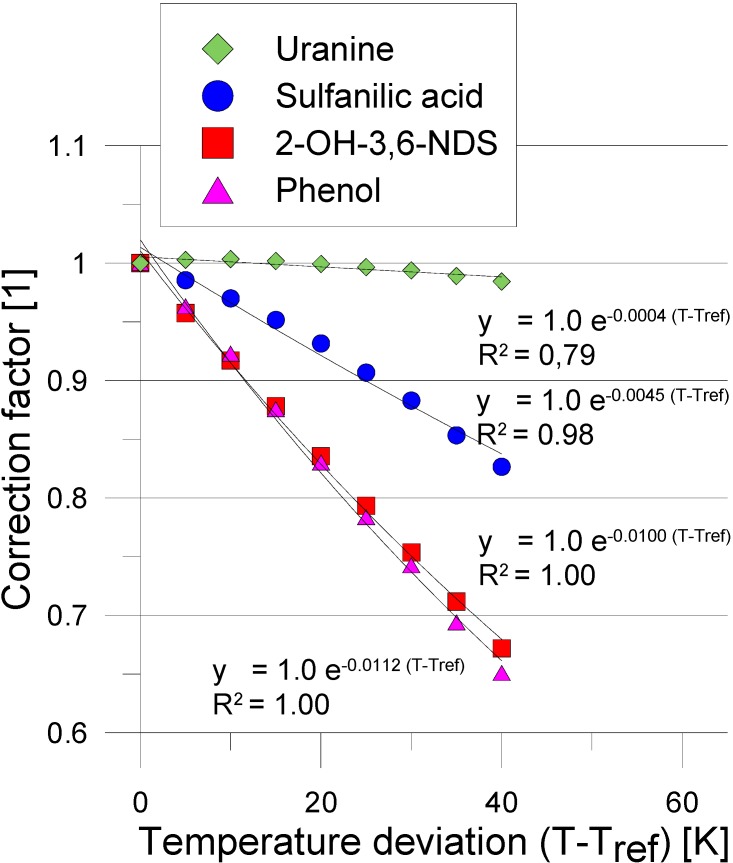
Temperature dependency of the reaction product of compound **7** (Sulfanilic acid). For comparison already tested tracers are shown. Uranine and 2-OH-3,6-NDS are conservative tracers as experimental reference while phenol and sulfanilic acid ae respective hydrolysis products from thermosensitive tracers.

The aim of the study was to conduct a physicochemical characterization of the synthesized compounds with focus on their suitability as thermosensitive tracers (TSTs) in geothermal reservoirs. We examined the esterss and their hydrolysis products for their fluorescence spectrum and fluorescence intensities, water solubility, detection limit by fluorescence spectroscopy depending on the temperature, sorption behavior, pH and temperature dependence of their hydrolysis kinetics, and their chemical reactivity. Compound **7** was the most promising candidate in terms of its good fluorescent properties. It is important that the tracer and its hydrolysis reaction products have distinguishable excitation and emission wavelengths while maintaining their high water solubility. Based on the scan of compound **7** carried out at the fluorescence spectrophotometer (Cary Eclipse, Varian GmbH, Darmstadt, Germany) we observed fluorescence of only one product (sulfanilic acid, Excitation 248 nm/Emission 344 nm) and as desired no fluorescent of compound **7** itself. Similar behavior exhibited phenol acetate, which was studied previously. Fluorescence intensities are affected by temperature. This will have an impact on observed tracer recovery in the experiments leading to an underestimation of the cooling state of the georeservoir. Therefore, this temperature fluorescence relationship needs to be known and considered during the experiments (Figure 1). 

**Figure 2:**
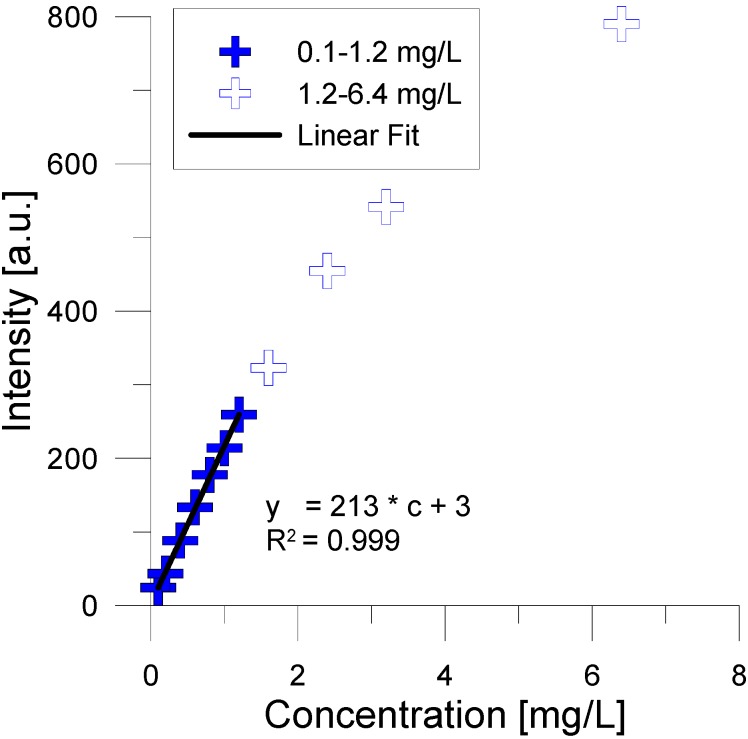
Linear range of fluorescence signal up to 1.2 mg/L.

Furthermore, tracer concentrations need to be adjusted to fall into a reliable and linear working range in the experiments. From a series of experiments with increasing sulfanilic acid concentrations it was obtained that above 1.2 mg/L the linear relationship breaks down (Figure 2). 

**Figure 3:**
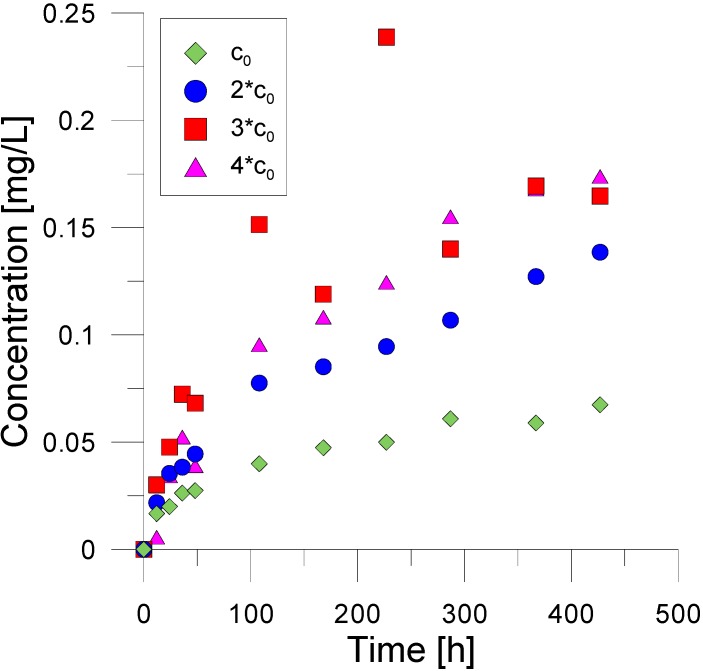
Results from a multi batch experiments with different starting concentrations of compound **7** for pH 6 and fluid temperature of 90 °C.

The comparison of phenol acetate with compound **7** revealed a higher stability under comparable pH and temperature conditions (pH ~ 6.5 and fluid temperature of 60 °C). This agrees with earlier made suggestions on target tracer design for geothermal applications based on esters. While a substantial amount of phenol acetate was consumed within a residence time of 2 h (half-life of phenol acetate under these conditions is ~23 h), virtually no consumption was observed for compound **7** during that time interval. A further examination at higher temperatures allowed the estimation of the half-lives and hence an examination of the reaction kinetics. Four experiments were performed in parallel with different dilution (i*c0, with i = 1, 2, 3, 4; Figure 3).

**Figure 4:**
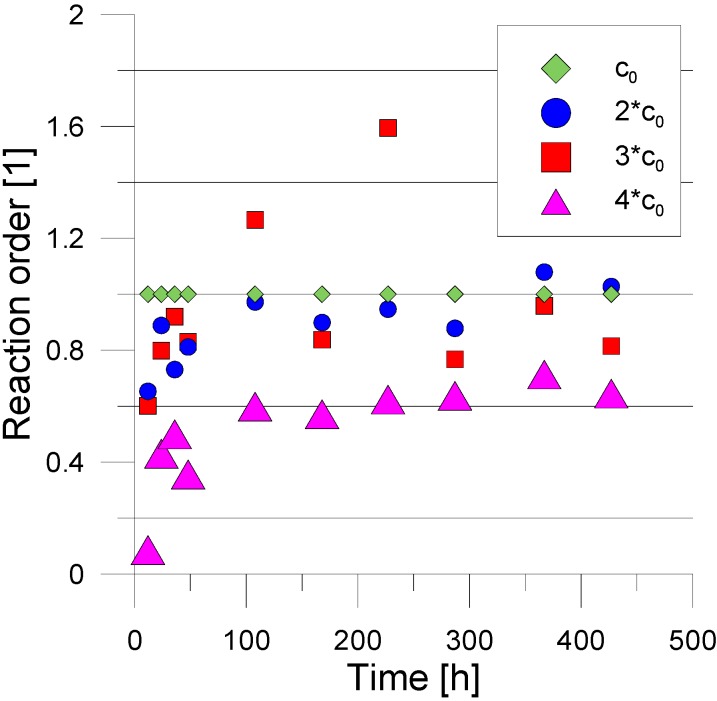
Reaction order for the different starting concentrations. The concentration changes for c_0_ is used as reference. For the analysis of the delay rate Equation (1) the values at *t* = 0, 108 and 227 h are used. The data for 3*c_0_ is excluded due to the high fluctuations.

The lowest dilution is c_0_. Initially, from the obtained concentrations the 1st order reaction was determined (Figure 4). To improve data quality of kinetic investigations, Guggenheim’s method [16] was applied in the analysis following:

(1) $$k=\frac{1}{\Delta t}\ln\left(\frac{c_{2}-c_{1}}{c_{3}-c_{2}}\right)$$

where *k* is the reaction rate, *c_k_* is the concentration at 3 output times and Δ*t* is a fixed time interval between the measurements (Figure 3). For ambient conditions with pH = 6 and a temperature of 90 °C a decay rate of 0.012 ± 0.001 1/h was obtained. This corresponds to an half live of 56 ± 7 h for the given pH and temperature. This already makes it a practical tracer for push pull experiments on the injection well of a doublet geothermal plant.

## 3. Experimental

### 3.1. General Information

All chemicals, reagents, and solvents were used as received from commercial sources without further purification. ^1^H-NMR and ^13^C-NMR spectra were recorded in CDCl_3_ on a 300 MHz liquid state Bruker spectrometer. The splitting patterns are annotated as follows: s (singlet), d (doublet), t (triplet), q (quadruplet), quint. (quintuplet), sext. (sextuplet), and m (multiplet). The products were also confirmed by ESI-MS (instrumentation is described in Nödler *et al.* [17]). Column chromatography was carried out on glass columns of different sizes packed with silica gel: Merck 60 (0.035–0.070 mm). 

### 3.2. Synthesis: General Procedure of Preparing Esters Containing Sulfonic Groups **1**–**18**

The primary alcoholic or phenolic sulfonic acid (2.0 mmol) is suspended in ethyl acetate (50 mL) and then an excess (2.28–2.4 mmol) of acid chloride is added. After the reaction suspension had been stirred intensively for 10 min at room temperature triethylamine (4 mmol) is added. The mixture was stirred overnight and then the solvent is removed under reduced pressure. The crude product was washed several times with ethyl acetate and subsequently purified by column chromatography (hexane/ethyl acetate, 1:5 and then methanol) or crystallized from methanol.

*3-(2,2-Dimethylpropanoyloxy)propane-1-sulfonic acid* (**1**). Yield: 0.38 g (85%). Yellow oil. ^1^H-NMR (300 MHz, CDCl_3_): δ, ppm, 4.14 (t, *J* = 6.4 Hz, 2H), 2.91–2.85 (m, 2H), 2.18–2.07 (m, 2H), 1.14 (s, 9H); ^13^C-NMR (300 MHz, CDCl_3_) δ, ppm, 178.44, 63.21, 48.26, 38.69, 27.13, 24.72. ESI-MS: *m*/*z* = 223 [M−H]^−^.

*3-(2-Ethylbutanoyloxy)propane-1-sulfonic acid* (**2**). Yield: 0.38 g (80%). Orange oil. ^1^H-NMR (300 MHz, CDCl_3_): δ, ppm, 4.17 (t, *J* = 6.4 Hz, 2H), 2.95–2.90 (m, 2H), 2.44–2.34 (m, 1H), 2.24–2.09 (m, 2H), 1.69–1.42 (m, 4H), 0.84 (t, *J* = 7.2 Hz, 6H); ^13^C-NMR (300 MHz, CDCl_3_) δ, ppm, 175.69, 62.83, 48.91, 48.32, 40.65, 24.97, 12.07. ESI-MS: *m*/*z* = 237 [M−H]^−^.

*4-(2,2-Dimethylpropanoyloxy)benzenesulfonic acid* (**3**). Yield: 0.51 g (99%). Yellow oil. ^1^H-NMR (300 MHz, CDCl_3_): δ, ppm, 7.86 (d, *J* = 8.7 Hz, 2H), 7.02 (d, *J* = 8.7 Hz, 2H), 1.31 (s, 9H); ^13^C-NMR (300 MHz, CDCl_3_) δ, ppm, 176.80, 152.18, 142.56, 127.33, 121.22, 39.05, 27.03. ESI-MS: *m*/*z* = 257 [M−H]^−^.

*4-(2-Ethylbutanoyloxy)benzenesulfonic acid* (**4**). Yield: 0.52 g (96%). Yellow oil.^1^H-NMR (300 MHz, CDCl_3_): δ, ppm, 7.89 (d, *J* = 8.7 Hz, 2H), 7.05 (d, *J* = 8.7 Hz, 2H), 2.47–2.38 (m, 1H), 1.83–1.54 (m, 4H), 0.99 (t, *J* = 7.5 Hz, 6H); ^13^C-NMR (300 MHz, CDCl_3_) δ, ppm, 174.27, 151.81, 142.57, 127.37, 121.29, 48.96, 25.11, 11.92. ESI-MS: *m*/*z* = 271 [M−H]^−^.

*4-(Acetyloxy)benzenesulfonic acid* (**5**). Yield: 0.40 g (92%). Brown oil. ^1^H-NMR (300 MHz, CDCl_3_): δ, ppm, 7.85 (d, *J* = 8.7 Hz, 2H), 7.05 (d, *J* = 8.7 Hz, 2H), 2.25 (s, 3H); ^13^C-NMR (300 MHz, CDCl_3_) δ, ppm, 168.92, 151.58, 142.63, 127.29, 121.21, 21.14. ESI-MS: *m*/*z* = 215 [M−H]^−^.

*4-(Pentanoyloxy)benzenesulfonic acid* (**6**). Yield: 0.44 g (86%). Yellow oil. ^1^H-NMR (300 MHz, CDCl_3_): δ, ppm, 7.79 (d, *J* = 8.7 Hz, 2H), 7.00 (d, *J* = 8.7 Hz, 2H), 2.47 (t, *J* = 7.5 Hz, 2H), 1.64 (quint., *J* = 7.5 Hz, 2H), 1.35 (sext., *J* = 7.5 Hz, 2H), 0.99 (t, *J* = 7.4 Hz, 3H); ^13^C-NMR (300 MHz, CDCl_3_) δ, ppm, 171.71, 151.55, 142.62, 127.10, 121.13, 33.93, 26.79, 22.09, 13.62. ESI-MS: *m*/*z* = 257 [M−H]^−^.

*4-(Benzoyloxy)benzenesulfonic acid* (**7**). Yield: 0.54 g (98%). White powder. M.p. 120 °C. ^1^H-NMR (300 MHz, CDCl_3_): δ, ppm, 8.15 (d, *J* = 7.2 Hz, 2H), 7.93 (d, *J* = 8.7 Hz, 2H), 7.61 (t, *J* = 7.5 Hz, 1H), 7.51–7.45 (m, 2H), 7.20 (d, *J* = 9.0 Hz, 2H); ^13^C-NMR (300 MHz, CDCl_3_) δ, ppm, 164.82, 151.95, 142.96, 133.68, 130.12, 129.24, 128.56, 127.47, 121.40. ESI-MS: *m*/*z* = 277 [M−H]^−^.

*4-(4-Methylbenzoyloxy)benzenesulfonic acid* (**8**). Yield: 0.56 g (96%). White powder. M.p. 88 °C. ^1^H-NMR (300 MHz, CDCl_3_): δ, ppm, 7.98 (d, *J* = 8.4 Hz, 2H), 7.85 (d, *J* = 8.7 Hz, 2H), 7.22 (d, *J* = 8.1 Hz, 2H), 7.13 (d, *J* = 8.7 Hz, 2H), 2.37 (s, 3H); ^13^C-NMR (300 MHz, CDCl_3_) δ, ppm, 164.63, 151.78, 144.39, 142.71, 130.32, 129.93, 129.09, 127.17, 121.26, 21.67. ESI-MS: *m*/*z* = 291 [M−H]^−^.

*4-(2,2-Dimethylpropanoyloxy)naphthalene-1-sulfonic acid* (**9**). Yield: 0.61 g (99%). Pink powder. M.p. 133 °C. ^1^H-NMR (300 MHz, CDCl_3_): δ, ppm, 8.85 (d, *J* = 8.7 Hz, 1H), 8.07 (d, *J* = 7.8 Hz, 1H), 7.74 (d, *J* = 8.1 Hz, 1H), 7.48 (td, *J* = 6.9, 1.8 Hz, 1H), 7.49 (td, *J* = 6.6, 1.2 Hz, 1H), 7.03 (d, *J* = 7.8 Hz, 1H), 1.37 (s, 9H); ^13^C-NMR (300 MHz, CDCl_3_) δ, ppm, 176.45, 148.38, 138.90, 130.15, 127.22, 127.02, 126.91, 126.20, 125.07, 120.78, 116.5, 39.31, 27.16. ESI-MS: *m*/*z* = 307 [M−H]^−^.

*4-(2-Ethylbutanoyloxy)naphthalene-1-sulfonic acid* (**10**). Yield: 0.61 g (95%). Yellow powder. M.p. 118 °C. ^1^H-NMR (300 MHz, CDCl_3_): δ, ppm, 8.94 (d, *J* = 8.1 Hz, 1H), 8.16 (d, *J* = 7.8 Hz, 1H), 7.88 (d, *J* = 8.4 Hz, 1H), 7.58 (td, *J* = 6.9, 1.5 Hz, 1H), 7.49 (td, *J* = 6.9, 1.2 Hz, 1H), 7.15 (d, *J* = 7.8 Hz, 1H), 2.66–2.56 (m, 1H), 1.95–1.81 (m, 2H), 1.80–1.69 (m, 2H), 1.08 (t, *J* = 7.4 Hz, 6H); ^13^C-NMR (300 MHz, CDCl_3_) δ, ppm, 174.23, 148.33, 138.91, 130.40, 127.35, 127.16, 127.13, 126.29, 125.27, 121.03, 116.26, 49.19, 25.12, 12.05. ESI-MS: *m*/*z* = 321 [M−H]^−^.

*4-(Acetyloxy)naphthalene-1-sulfonic acid* (**11**). Yield: 0.52 g (99%). Brown oil. ^1^H-NMR (300 MHz, CDCl_3_): δ, ppm, 8.91 (d, *J* = 8.4 Hz, 1H), 8.13 (d, *J* = 7.8 Hz, 1H), 7.83 (d, *J* = 8.4 Hz, 1H), 7.55 (td, *J* = 6.9, 1.2 Hz, 1H), 7.47 (td, *J* = 6.9, 1.2 Hz, 1H), 7.15 (d, *J* = 7.8 Hz, 1H), 2.40 (s, 3H); ^13^C-NMR (300 MHz, CDCl_3_) δ, ppm, 169.06, 148.22, 139.20, 130.36, 127.20, 127.19, 127.15, 126.42, 125.28, 121.10, 116.35, 20.93. ESI-MS: *m*/*z* = 265 [M−H]^−^.

*4-(Benzoyloxy)naphthalene-1-sulfonic acid* (**12**). Yield: 0.64 g (98%). Pink powder. M.p. 183 °C. ^1^H-NMR (300 MHz, CDCl_3_): δ, ppm, 8.90 (d, *J* = 8.4 Hz, 1H), 8.21 (d, *J* = 7.2 Hz, 2H), 8.14 (d, *J* = 7.8 Hz, 1H), 7.85 (d, *J* = 8.4 Hz, 1H), 7.64–7.58 (m, 1H), 7.54–7.38 (m, 4H), 7.23 (d, *J* = 7.5 Hz, 1H); ^13^C-NMR (300 MHz, CDCl_3_) δ, ppm, 164.64, 148.30, 139.23, 133.73, 130.27, 130.00, 128.76, 128.55, 127.22, 127.10, 127.07, 126.34, 125.14, 121.07, 116.40. ESI-MS: *m*/*z* = 327 [M−H]^−^.

*4-(4-Methylbenzoyloxy)naphthalene-1-sulfonic acid* (**13**). Yield: 0.65 g (96%). Pink powder. M.p. 204 °C. ^1^H-NMR (300 MHz, CDCl_3_): δ, ppm, 8.89 (d, *J* = 8.4 Hz, 1H), 8.14 (d, *J* = 8.1 Hz, 1H), 8.09 (d, *J* = 8.4 Hz, 2H), 7.84 (d, *J* = 8.4 Hz, 1H), 7.51 (td, *J* = 6.9, 1.5 Hz, 1H), 7.41 (td, *J* = 6.9, 1.2 Hz, 1H), 7.27 (d, *J* = 7.8 Hz, 2H), 7.22 (d, *J* = 8.1 Hz, 1H), 2.39 (s, 3H); ^13^C-NMR (300 MHz, CDCl_3_) δ, ppm, 164.70, 148.40, 144.66, 139.11, 130.25, 130.05, 129.26, 127.29, 127.07, 127.03, 126.29, 125.98, 125.16, 121.13, 116.43, 21.70. ESI-MS: *m*/*z* = 341 [M−H]^−^.

*4-(3-Phenylpropanoyloxy)naphthalene-1-sulfonic acid* (**14**). Yield: 0.28 g (40%). Brown powder. M.p. 93 °C. ^1^H-NMR (300 MHz, CDCl_3_): δ, ppm, 8.91 (d, *J* = 8.7 Hz, 1H), 8.14 (d, *J* = 7.8 Hz, 1H), 7.60–7.51 (m, 2H), 7.42 (d, *J* = 7.8 Hz, 1H), 7.38–7.25 (m, 5H), 7.09 (d, *J* = 7.8 Hz, 1H), 3.13 (t, *J* = 6.6 Hz, 2H), 3.04 (t, *J* = 6.9 Hz, 2H); ^13^C-NMR (300 MHz, CDCl_3_) δ, ppm, 170.96, 148.18, 139.80, 138.99, 130.32, 128.56, 128.33, 127.18, 127.09, 127.04, 126.44, 126.34, 125.28, 121.08, 116.24, 35.95, 31.95. ESI-MS: *m*/*z* = 355 [M−H]^−^.

*4-(2,2-Dimethylpropanoyloxy)-7-(phenylamino)naphthalene-2-sulfonic acid* (**15**). Yield: 0.64 g (80%). Gray powder. M.p. 112 °C. ^1^H-NMR (300 MHz, CDCl_3_): δ, ppm, 7.93 (s, 1H), 7.54 (d, *J* = 9.0 Hz, 1H), 7.43 (d, *J* = 2.1 Hz, 1H), 7.34–7.29 (m, 2H), 7.22–7.12 (m, 4H), 6.86 (t, *J* = 6.8 Hz, 1H), 1.34 (s, 9H); ^13^C-NMR (300 MHz, CDCl_3_) δ, ppm, 176.38, 146.80, 142.82, 142.36, 142.02, 134.96, 129.01, 122.13, 121.95, 121.37, 121.19, 121.05, 118.45, 112.45, 110.55, 39.24, 27.10. ESI-MS: *m*/*z* = 398 [M−H]^−^.

*4-(2-Ethylbutanoyloxy)-7-(phenylamino)naphthalene-2-sulfonic acid* (**16**). Yield: 0.64 g (78%). Brown powder. M.p. 97 °C. ^1^H-NMR (300 MHz, CDCl_3_): δ, ppm, 7.96 (s, 1H), 7.60 (d, *J* = 9.0 Hz, 1H), 7.44 (d, *J* = 2.4 Hz, 1H), 7.32 (td, *J* = 5.0, 2.0 Hz, 2H), 7.21–7.13 (m, 4H), 6.88 (t, *J* = 7.6 Hz, 1H), 2.54–2.45 (m, 1H), 1.81–1.67 (m, 2H), 1.66–1.54 (m, 2H), 0.98 (t, *J* = 7.5 Hz, 6H); ^13^C-NMR (300 MHz, CDCl_3_) δ, ppm, 174.11, 146.62, 142.83, 142.41, 142.02, 135.02, 129.06, 122.11, 121.46, 121.37, 121.19, 121.03, 118.57, 112.61, 110.55, 49.02, 25.04, 11.96. ESI-MS: *m*/*z* = 412 [M−H]^−^.

*4-(Benzoyloxy)-7-(phenylamino)naphthalene-2-sulfonic acid* (**17**). Yield: 0.71 g (85%). Brown powder. M.p. 94 °C. ^1^H-NMR (300 MHz, CDCl_3_): δ, ppm, 8.17 (dd, *J* = 8.6, 1.4 Hz, 2H), 8.02 (s, 1H), 7.65 (d, *J* = 8.7 Hz, 1H), 7.59 (d, *J* = 7.5 Hz, 1H), 7.50–7.45 (m, 4H), 7.28 (dd, *J* = 9.0, 2.1 Hz, 1H), 7.23–7.12 (m, 4H), 6.88 (t, *J* = 7.0 Hz, 1H); ^13^C-NMR (300 MHz, CDCl_3_) δ, ppm, 164.68, 146.75, 142.89, 142.50, 142.05, 135.12, 133.62, 129.96, 129.08, 128.54, 127.99, 122,26, 122.09, 121.65, 121.43, 121.11, 118.50, 112.75, 110.69. ESI-MS: *m*/*z* = 418 [M−H]^−^.

*4-(4-Methylbenzoyloxy)-7-(phenylamino)naphthalene-2-sulfonic acid* (**18**). Yield: 0.71 g (82%). Gray powder. M.p. 181 °C. ^1^H-NMR (300 MHz, CDCl_3_): δ, ppm, 8.05 (d, *J* = 8.1 Hz, 2H), 8.01 (s, 1H), 7.65 (d, *J* = 9.0 Hz, 1H), 7.48 (d, *J* = 1.5 Hz, 1H), 7.46 (d, *J* = 2.4 Hz, 1H), 7.30–7.25 (m, 3H), 7.19 (d, *J* = 7.8 Hz, 2H), 7.17–7.13 (m, 2H), 6.88 (t, *J* = 6.9 Hz, 1H), 2.40 (s, 3H); ^13^C-NMR (300 MHz, CDCl_3_) δ, ppm, 164.73, 146.84, 144.50, 142.92, 142.42, 142.06, 135.09, 130.00, 129.25, 129.08, 126.18, 122.33, 122,20, 121.56, 121.44, 121.08, 118.48, 112.80, 110.75, 21.72. ESI-MS: *m*/*z* = 432 [M−H]^−^.

## 4. Conclusions

We have developed a very simple, inexpensive, nontoxic, and environmentally friendly method for the acylation of primary alcohols and phenols. The presence of the SO_3_H group in all synthesized esters ensures their good water solubility and thus their usefulness as tracers in georeservoirs. In particular, there is little information related to the synthesis of readily water soluble esters based on fluorescent molecules. We strongly feel that the study presented here will find numerous applications even beyond tracers for geothermal applications. There are many methods of ester preparation, but almost all of them do not work in case of reagents containing sulfonic groups. During our work we investigated a series of esterification methods, but only one fulfills the desired requirements, hence the efficient method we presented in this paper.
